# 169. Combining DRIP Score and Rapid Diagnostics for Improved Antibiotic Stewardship

**DOI:** 10.1093/ofid/ofac492.247

**Published:** 2022-12-15

**Authors:** Richard Ramirez, Matthew Sims

**Affiliations:** Oakland University William Beaumont School of Medicine, Ypsilanti, Michigan; Beaumont Health, Royal Oak, Michigan

## Abstract

**Background:**

Analysis of broad-spectrum antibiotic use in pneumonia revealed that 60% of patients were overtreated, highlighting the need for effective antibiotic stewardship practices.

Systems such as the Drug Resistance in Pneumonia (DRIP) score select patients who are more likely to need broad spectrum antibiotics but still leads to overtreatment as it does not target specific pathogens. Rapid diagnostics such as the Unyvero Lower Respiratory Tract Panel (LRTP) can identify specific pathogens to narrow antibiotic use even further when combined with the DRIP score.

**Methods:**

Using an existing patient pool from a clinical trial of the LRTP (NCT01922024) a DRIP score was determined for each patient. When data elements of the DRIP score were unavailable a DRIP_max_ and DRIP_min_ were calculated assuming all missing elements were positive or negative respectively. The sensitivity and specificity of the DRIP score vs culture and LRTP were determined. An algorithm for antibiotic selection based on the results of the DRIP score combined with the LRTP was applied to each patient (Fig 1) .

**Figure 1.**
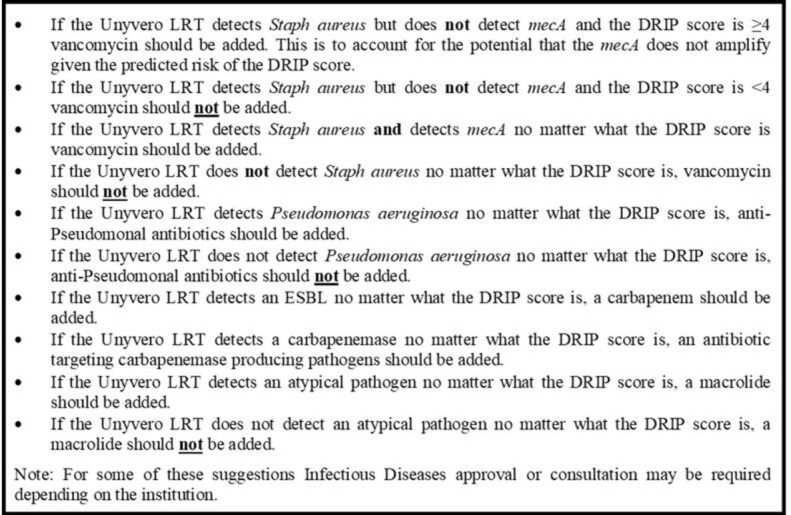
Antibiotic Selection Algorithm for DRIP Score and Unyvero Lower Respiratory Tract Panel

**Results:**

The sensitivity of the DRIP score vs culture in this population was 91.2% and the specificity was 65.1%. DRIP score vs culture + LRTP combined had a sensitivity of 86.6% and a specificity of 66.3%., the lower sensitivity was mainly due to *Stenotrophomonas maltophilia* Applying the algorithm to each patient based on the DRIP score and the LRTP improved antibiotic choices by reducing broad spectrum antibiotic and by more selectively targeting the identified pathogens earlier and eliminating use of multiple empiric antibiotics.

**Conclusion:**

Using an antibiotic stewardship algorithm combining DRIP score with LRTP rapid diagnostic data, which results in 5 hours, can lead to improved prediction of the presence of drug resistant pathogens and aid in narrowing antibiotics. The LRTP compensates for the DRIP score only predicting presence of antibiotic resistant pathogens, not specific pathogens. The DRIP score can compensate for the LRTP only having limited antibiotic resistance markers on panel and not supplying phenotypic antibiotic resistance testing. Further study using a prospectively collected cohort with antibiotic adjustment in real time is needed for validation of our results.

**Disclosures:**

**Matthew Sims, MD PhD**, Astra Zeneca: Grant/Research Support|ContraFect: Grant/Research Support|Crestone: Grant/Research Support|Diasorin Molecular LLC: Grant/Research Support|Epigenomics Inc: Grant/Research Support|EUROIMMUN US: Grant/Research Support|Finch Theraputics: Grant/Research Support|Genentech USA Inc: Grant/Research Support|Janssen Research and Development LLC: Grant/Research Support|Kinevant Sciences GmBH: Grant/Research Support|Leonard-Meron Biosciences: Grant/Research Support|Lysovant: Grant/Research Support|Merck: Grant/Research Support|OpGen: Grant/Research Support|Prenosis: Advisor/Consultant|Prenosis: Grant/Research Support|Qiagen: Grant/Research Support|Regeneron Pharmaceuticals: Grant/Research Support|Seres Therapeutics Inc: Grant/Research Support|Shire: Grant/Research Support|Summit Therapeutics: Grant/Research Support.

